# Associations between disrupted functional brain network topology and cognitive impairment in patients with rectal cancer during chemotherapy

**DOI:** 10.3389/fonc.2022.927771

**Published:** 2022-11-24

**Authors:** Yesong Guo, Siwen Liu, Fei Yan, Na Yin, Jie Ni, Chenchen Li, Xuan Pan, Rong Ma, Jianzhong Wu, Shengwei Li, Xiaoyou Li

**Affiliations:** ^1^ Department of Radiotherapy, Jiangsu Cancer Hospital & Jiangsu Institute of Cancer Research & The Affiliated Cancer Hospital of Nanjing Medical University, Nanjing, China; ^2^ Research Center for Clinical Oncology, Jiangsu Cancer Hospital & Jiangsu Institute of Cancer Research & The Affiliated Cancer Hospital of Nanjing Medical University, Nanjing, China; ^3^ Department of Oncology, Jiangsu Cancer Hospital & Jiangsu Institute of Cancer Research & The Affiliated Cancer Hospital of Nanjing Medical University, Nanjing, China; ^4^ Department of Radiology, Jiangsu Cancer Hospital & Jiangsu Institute of Cancer Research & The Affiliated Cancer Hospital of Nanjing Medical University, Nanjing, China; ^5^ Department of Anorectal, Yangzhou Traditional Chinese Medicine Hospital Affiliated to Nanjing University of Chinese Medicine, Yangzhou, China

**Keywords:** rectal cancer, resting-state functional magnetic resonance imaging, graph theory analysis, chemotherapy, cognitive impairment

## Abstract

**Introduction:**

Cognitive impairment has been identified in patients with non-central nervous system cancer received chemotherapy. Chemotherapy-induced changes in the brain are considered as the possible causes of the cognitive deficits of patients. This study aimed to explore chemotherapy-related functional brain changes and cognitive impairment in rectal cancer (RC) patients who had just finished chemotherapy treatment.

**Methods:**

In this study, RC patients after chemotherapy (on the day patients received the last dose of chemotherapy) (n=30) and matched healthy controls (HCs) (n=30) underwent cognitive assessments, structural magnetic resonance imaging (MRI) and resting-state functional MRI. The functional brain networks were constructed by thresholding the partial correlation matrices of 90 brain regions in the Anatomical Automatic Labeling template and the topologic properties were evaluated by graph theory analysis. Moreover, correlations between altered topological measures and scores of cognitive scales were explored in the patient group.

**Results:**

Compared with HCs, RC patients had lower scores of cognitive scales. The functional brain network had preserved small-world topological features but with a tendency towards higher path length in the whole network. In addition, patients had decreased nodal global efficiency (E_glo(i)_) in the left superior frontal gyrus (dorsolateral), superior frontal gyrus (orbital part), inferior frontal gyrus (opercular part), inferior frontal gyrus (triangular part) and right inferior frontal gyrus (triangular part). Moreover, values of E_glo(i)_ in the superior and inferior frontal gyrus were positively associated with cognitive function in the patient group.

**Conclusion:**

These results suggested that cognitive impairment was associated with disruptions of the topological organization in functional brain networks of RC patients who had just finished chemotherapy, which provided new insights into the pathophysiology underlying acute effects of chemotherapy on cognitive function.

## Introduction

Colorectal cancer (CRC) is the third most common malignancies (1) and second highest cause of cancer related deaths worldwide (2). In addition, rectal cancer (RC) accounts for approximately 30% of all CRC cases (3), and 55% RC patients are diagnosed in the stage of II/III (4). The standard treatment for locally advanced RC (LARC) (T3-T4 and/or N+ or any T with N+) includes neoadjuvant chemoradiotherapy (nCRT) and total mesorectal excision (TME) (5). With the development of surgical techniques, nCRT and adjuvant chemotherapy, 5-year local recurrence rates of RC after resection have decreased to 5%-10% (6). However, the combination of nCRT and TME has not improved the survival rate (7). Distant metastasis remains the major cause of treatment failure among patients with LARC and the incidence of distant metastasis at 10 years is 25-28% (8). To reduce the risk of distant metastasis and increase the survival rate, adjuvant chemotherapy has been recommended LARC patients after TME (9). It has been reported that the combination of oxaliplatin and nCRT can lead to increased pathological complete remission rates in patients with LARC (10). Therefore, adjuvant chemotherapy may play an important role in the treatment of patients with higher relapse risk, particularly in those with no or lower responses to nCRT.

When compared with preoperative chemoradiotherapy, significantly prolonged overall survival and improved pathological complete response rates were achieved in patients with stage T3 or T4 RC receiving the neoadjuvant regimen of fluorouracil, leucovorin, irinotecan and oxaliplatin (FOLFIRINOX) followed by chemoradiotherapy (11). When compared with the standard fluorouracil-based combined modality regimen, a disease-free survival benefit of adding oxaliplatin to fluorouracil-based nCRT and adjuvant chemotherapy was found in patients with RC (staged as cT3-4 or any node-positive disease) after TME surgery (12). In addition, higher 5-year overall survival, 3-year disease-free survival, pathological complete response rates and fewer distant metastases were detected in patients with stage II-III LARC who received capecitabine-based chemoradiotherapy when compared with the fluorouracil-based chemoradiotherapy (13). Therefore, both oxaliplatin and capecitabine can offer effective and convenient alternatives for LARC patients undergoing nCRT or adjuvant chemotherapy. However, chemotherapy-induced neurotoxicity on cognition has been acknowledged (14, 15) and chemotherapy-induced brain abnormalities related to cognitive dysfunction has been recognized (16, 17).

Resting-state functional magnetic resonance imaging (rs-fMRI) can reflect spontaneous neural activity through the fluctuations of regional blood-oxygen-level-dependent (BOLD) signals (18). It provides a new technique for exploring the pathological mechanism of cognitive impairment, which is characterized by impaired brain functional connectivity involved in the cognitive processing (19, 20). Changes of brain function and functional connectivity have been found in cancer patients receiving chemotherapy by rs-fMRI (21, 22). Chemotherapy-induced cognitive impairments were found to be related to the brain functional alterations in previous rs-fMRI studies (23, 24). Graph theory analysis has been applied to investigate the topological characteristics of functional brain network, which regards the brain as a set of regions (nodes) connected by functional connections (edges) (25, 26). Graph theory-based network analysis provides both global and local measures for the connectivity in the brain (27). Based on this advantage, graph theory analysis has also been widely used to explore the pathological mechanism of cognitive dysfunction with rs-fMRI data (28). To our knowledge, no study has been conducted to explore the neuropathological mechanism of chemotherapy-induced cognitive impairment in RC patients by graph theory analysis.

Based on the findings of previous studies, we proposed that the functional brain network of RC patients might exhibit changed topological features, which were associated with the acute effects of chemotherapy on cognitive function. Therefore, graph theory analysis was employed to explore the topological characteristics of the functional brain network in patients with RC who had just completed chemotherapy (on the day patients received the last dose of chemotherapy). Additionally, the relationships between altered topological measures and cognitive performance of patients were examined.

## Materials and methods

### Participants

This study was approved by the Medical Ethics Committee of Jiangsu Cancer Hospital & Jiangsu Institute of Cancer Research & The Affiliated Cancer Hospital of Nanjing Medical University. The sample size was estimated as follows: δ=(μ_D_-0)/, where δ was the effect size, μ_D_ was the difference in means between groups, 0 was the difference in means under the null hypothesis, and δ was the variability in the difference in means (29). In fMRI, μ_D_ and δ were typically normalized as percent signal change (i.e. 100×(*E*-*C*)/*C*, where *E*, patients and *C*, HCs) (29). However, it was difficult to estimate the appropriate sample size according to the effect size as other types of studies because of the relatively poor statistical effectiveness and the large heterogeneity among studies (30, 31). At present, there was no final conclusion on this issue. Thirion et al. suggested that more than 27 subjects might be appropriate (32). In this study, a total of 30 post-chemotherapy RC patients (be recruited on the day patients received the last dose of chemotherapy) and 30 sex-, age-, and education-matched healthy controls (HCs) were recruited. All participants were right-handed Han Chinese and written informed consent was obtained from each participant before participating in this study. All patients were diagnosed pathologically as rectal adenocarcinoma through surgery or enteroscopy and received Oxaliplatin and Capecitabine based chemotherapy regimen for 2-3 months. In addition, none of them had presence of central nervous system metastases and other cancer. The details about the demographic and clinical features of patients and HCs were described in **Table 1**.

**Table 1:** Demographic and clinical characteristics of participants.

Variables	RC (n=30)	HCs (n=30)	*t*/χ^2^	*P*
Age (years)	59.80 ± 8.62	58.07 ± 10.00	0.72	0.48^a^
Gender (M/F)	16/14	21/9	1.12	0.29^b^
Education level (years)	13.80 ± 1.54	14.10 ± 1.47	-0.77	0.44^a^
Cognitive function assessment				
Scores of MMSE	25.77 ± 1.61	26.77 ± 1.25	-2.68	0.0095^a^
Scores of MoCA	27.07 ± 0.87	27.67 ± 0.81	-2.78	0.0073^a^
Scores of FACT-Cog	98.03 ± 3.82	100.73 ± 3.13	-3.00	0.0040^a^
Disease stage (n)				
I	0			
II	1			
III	23			
IV	6			
Metastasis (n)				
No	8			
Lung	1			
Liver	16			
Intraperitoneal	4			
Pelvic	1			
Chemotherapy regimen (n)				
Oxaliplatin+Capecitabine	7			
Oxaliplatin+Capecitabine+Bevacizumab	23			

RC, rectal cancer; HCs, healthy controls. MMSE, Mini Mental State Exam; MoCA, Montreal Cognitive Assessment; FACT-Cog, Functional Assessment of Cancer Therapy-Cognitive Function. ^a^: P values were obtained by the method of two sample t-tests. ^b^: P value was obtained by the method of Chi-square test. P<0.05 was considered to be statistically significant.

The exclusion criteria were as follows: (1) history of radiation therapy; (2) major medical illnesses (e.g., severe cardiovascular, hepatic or renal, endocrine, hematological diseases and other severe digestive problems, etc.); (3) neurological or psychiatric illnesses (e.g., schizophrenia, emotional disorders like depression, brain trauma, stroke, loss of consciousness, epilepsy, Parkinson’s disease, Alzheimer’s disease, etc.); (4) alcohol or drug abuse; (5) any contraindication for MRI scanning.

### Assessment of cognitive function

On the day patients received the last dose of chemotherapy, the level of cognitive function of all subjects in this study was assessed by two professionally trained clinicians using the self-report questionnaires including Mini Mental State Exam (MMSE) (33), Montreal Cognitive Assessment (MoCA) (34) and Functional Assessment of Cancer Therapy-Cognitive Function (FACT-Cog) (35).

### MRI data acquisition, preprocessing and brain network construction

**Figure 1:**
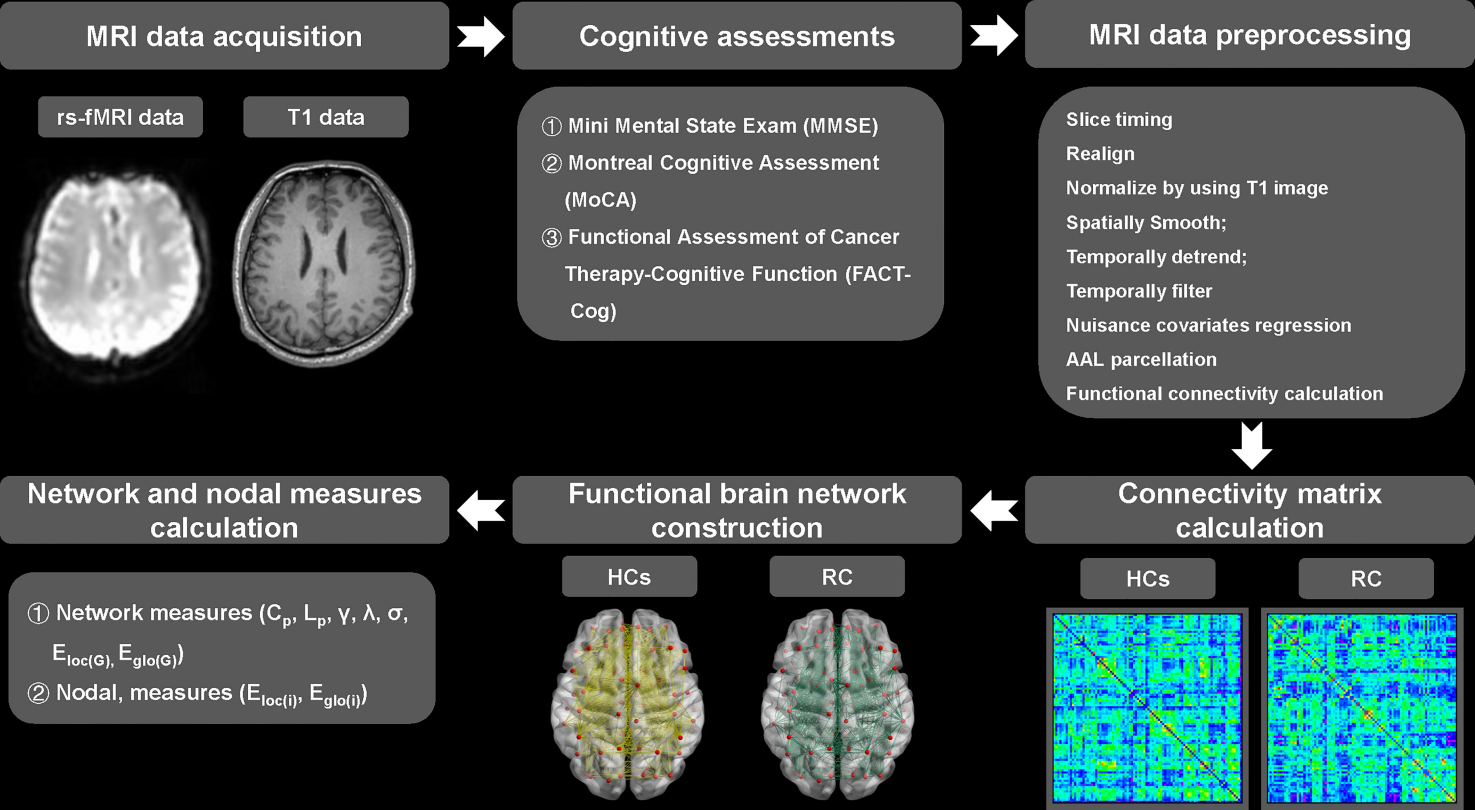
Pipeline of MRI data acquisition, preprocessing, functional brain network construction and topological measures calculation. RC, rectal cancer; HCs, healthy controls. γ, normalized clustering coefficient; λ, normalized characteristic path length; σ, small-worldness. C_p_, clustering coefficient of network; L_p_, characteristic path length of network; E_loc(G)_, local efficiency of network; E_glo(G)_, global efficiency of network.

On the day patients received the last dose of chemotherapy, T1 and rs-fMRI data of all participants were acquired with a 3.0 T Philips Aachieva scanner at Jiangsu Cancer Hospital & Jiangsu Institute of Cancer Research & The Affiliated Cancer Hospital of Nanjing Medical University. All MRI data were processed using the Data Processing Assistant for rs-fMRI advanced edition (DPARSF) (36). For the functional brain network, nodes were defined as 90 brain regions in divided by the Anatomical Automatic Labeling (AAL) template (37), whereas edges were defined as the functional connections between brain regions. The details about MRI parameters, steps of MRI data acquisition, preprocessing and brain network construction were presented in our previous study (38) and were illustrated in **Figure 1** (see **Supplementary materials**).

### Calculation of topological measures

The software of GRETNA was applied for the calculation of topological measures including network measures (small-world parameters involving clustering coefficient C_p_, characteristic path length L_p_, normalized clustering coefficient γ, normalized characteristic path length λ, small-worldness σ, and network efficiency involving local efficiency E_loc(G)_ and global efficiency E_glo(G)_, as well as nodal measures (local efficiency E_loc(i)_ and global efficiency E_glo(i)_) (39). The sparsity was defined as the ratio of the actual number of edges divided by the maximum possible number of edges in the network. Given no gold standard to select a single threshold, these measures were calculated with a wide range of sparsity thresholds (0.05-0.5) with an interval of 0.01 (40, 41). In addition, the area under the curve (AUC) value for each metric was calculated for sparsity thresholds from 0.05 to 0.5. The value of AUC was independent of single threshold selection and provided a summarized scalar for exploring the topological properties of functional brain network. Therefore, AUC has been proved to be highly sensitive for detecting the topological abnormalities of brain disorders.

### Statistical analysis

In study, statistical analyses were performed using the software of SPSS version 23. Group-differences of demographic and clinical data were compared by two-sample *t*-test for continuous variables and Chi-square test for categorical variables. Between-group differences in the topological measures and their AUC of the functional brain network were evaluated by two-sample *t*-test. In addition, FDR method was performed to correct for multiple comparisons of the AUC of nodal measures including E_loc(i)_ and E_glo(i)_ separately (90 brain regions were compared between groups; multiple sets=90; *P*_i_≤ 0.05*i/90, all tests associated with *P*_1_…*P*_i_ were declared significant). Moreover, the associations between altered measures of the functional brain network and the cognitive scale scores were explored in RC patients by the method of *Pearson’s* correlation analysis. *P*<0.05 was considered statistically significant.

## Results

### Differences of demographic and clinical characteristics

As shown in **Table 1**, there were no differences in the age, gender and education level between RC patients and HCs. However, compared to HCs, RC patients had a significantly lower scores on MMSE, MoCA and FACT-Cog, which suggested that patients had lower level of subjective cognitive functioning at the end of chemotherapy treatment.

### Differences of small-world properties and network measures

**Figure 2:**
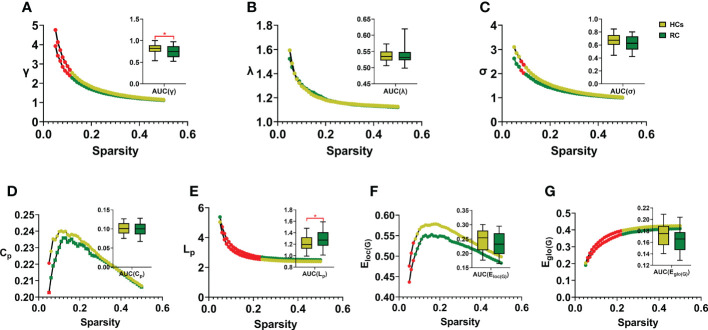
Comparison of small-world properties and network measures between groups. RC, rectal cancer; HCs, healthy controls. AUC, area under curve. γ, normalized clustering coefficient; λ, normalized characteristic path length; σ, small-worldness **(A-C)**. C_p_: clustering coefficient of network; L_p_, characteristic path length of network; E_loc(G)_, local efficiency of network; E_glo(G)_, global efficiency of network **(D-G)**. Red dots and stars indicated statistical significance *P* < 0.05. Two sample *t*-tests were employed for statistical analysis and *P* value less than 0.05 was considered significant. * indicated statistical significance.

In this study, the small-world parameters (γ, λ and σ) and network measure (C_p_, L_p_, E_loc(G)_ and E_glo(G)_) of functional brain network were calculated at a wide range of sparsity (0:05-0:5 with 0.01 increments) (**Table 2**; **Figure 2**). Over the whole sparsity range, both RC patients and HCs met the criteria of small-worldness (σ>1). Compared with HCs, RC patients had decreased γ (in the sparsity range of 0.05-0.11) and σ (0.08-0.09), whereas no differences of λ were found between groups. In addition, RC patients had decreased C_p_ (at the sparsity range of 0.05), L_p_ (0.06-0.23), E_loc(G)_ (0.05-0.07) and E_glo(G)_ (0.06-0.22) when compared with HCs. Moreover, AUC for each metric was calculated. The AUC of γ and L_p_ were lower in the patient group than those of HCs.

**Table 2:** Comparison of the AUC of small-world and network measures between groups.

	RC (n=30)	HCs (n=30)	*t*	*P*
Small-world				
γ	0.75 ± 0.14	0.82 ± 0.11	2.08	0.04
λ	0.54 ± 0.023	0.54 ± 0.019	-0.06	0.95
σ	0.62 ± 0.12	0.68 ± 0.10	1.92	0.06
Network measures				
C_p_	0.10 ± 0.018	0.10 ± 0.016	0.35	0.73
L_p_	1.29 ± 0.14	1.21 ± 0.13	-2.21	0.03
E_loc(G)_	0.23 ± 0.039	0.25 ± 0.035	1.08	0.29
E_glo(G)_	0.16 ± 0.019	0.17 ± 0.020	1.88	0.064

RC, rectal cancer; HCs, healthy controls. AUC, area under curve. γ, normalized clustering coefficient; λ, normalized characteristic path length; σ, small-worldness. C_p_, clustering coefficient of network; L_p_, characteristic path length of network; E_loc(G)_, local efficiency of network; E_glo(G)_, global efficiency of network. Uncorrected P values were obtained by the method of two sample t-tests. P<0.05 indicated statistically significant differences.

### Differences in the AUC of nodal measures

**Figure 3:**
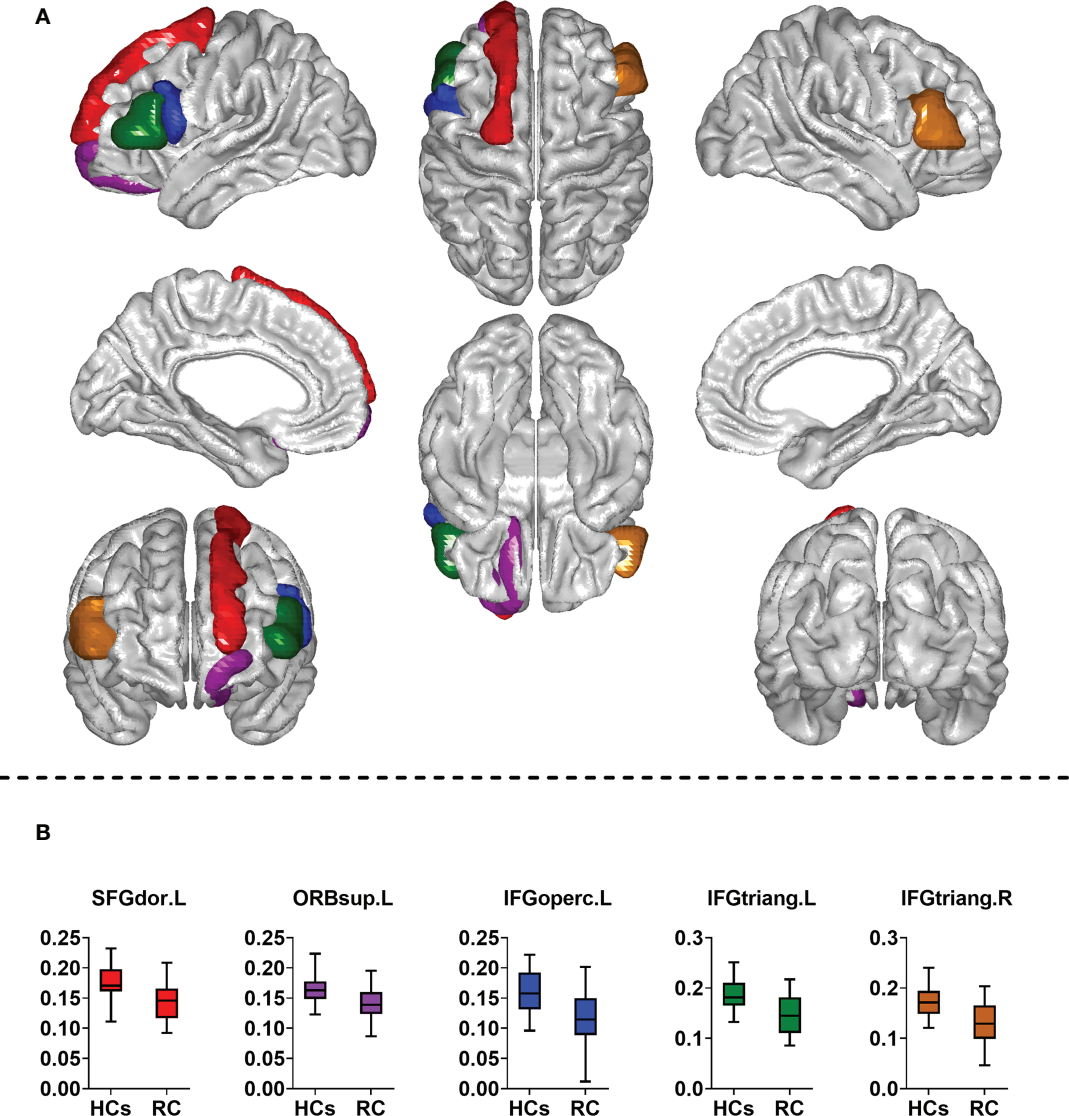
Comparison of nodal measures between groups. RC, rectal cancer; HCs, healthy controls. SFGdor. L, left superior frontal gyrus (dorsolateral); ORBsup.L, left superior frontal gyrus (orbital part); IFGoperc.L, left inferior frontal gyrus (opercular part); IFGtriang.L, left inferior frontal gyrus (triangular part); IFGtriang. R, right inferior frontal gyrus (triangular part). Two sample *t*-tests were employed for statistical analysis. In addition, false discovery rate (FDR) method was used to correct for multiple comparisons. Corrected *P* < 0.05 indicated statistically significant differences. **(A)** Brain regions with altered nodal global efficiency; **(B)** Comparison of nodal global efficiency between groups.

The between-group comparison revealed that the RC patients exhibited decreased E_glo(i)_ in the left superior frontal gyrus (dorsolateral), superior frontal gyrus (orbital part), inferior frontal gyrus (opercular part), inferior frontal gyrus (triangular part) and right inferior frontal gyrus (triangular part) when compared with HCs (**Table 3**; **Figure 3**). However, no differences of E_loc(i)_ were found between groups.

**Table 3:** Brain regions showing differences in the AUC of nodal measures between groups.

Nodal measures	Brain regions	RC (n=30)	HCs (n=30)	*t*	*P*
E_loc(i)_	No regions showing differences				
E_glo(i)_	Left superior frontal gyrus (dorsolateral)	0.14 ± 0.032	0.18 ± 0.029	-4.09	0.00014
	Left superior frontal gyrus (orbital part)	0.14 ± 0.027	0.17 ± 0.022	-3.76	0.00040
	Left inferior frontal gyrus (opercular part)	0.12 ± 0.049	0.16 ± 0.035	-4.14	0.00012
	Left inferior frontal gyrus (triangular part)	0.15 ± 0.039	0.19 ± 0.031	-4.57	0.000026
	Right inferior frontal gyrus (triangular part)	0.13 ± 0.040	0.17 ± 0.032	-4.39	0.000048

RC, rectal cancer; HCs, healthy controls. AUC, area under curve. E_loc(i)_, nodal local efficiency; E_glo(i)_, nodal global efficiency. FDR corrected P values were obtained by the method of two sample t-tests. In addition, false discovery rate (FDR) method was used to correct for multiple comparisons. Corrected P<0.05 indicated statistically significant differences.

### Associations between altered topological parameters and scores of cognitive scales in the patient group

**Figure 4:**
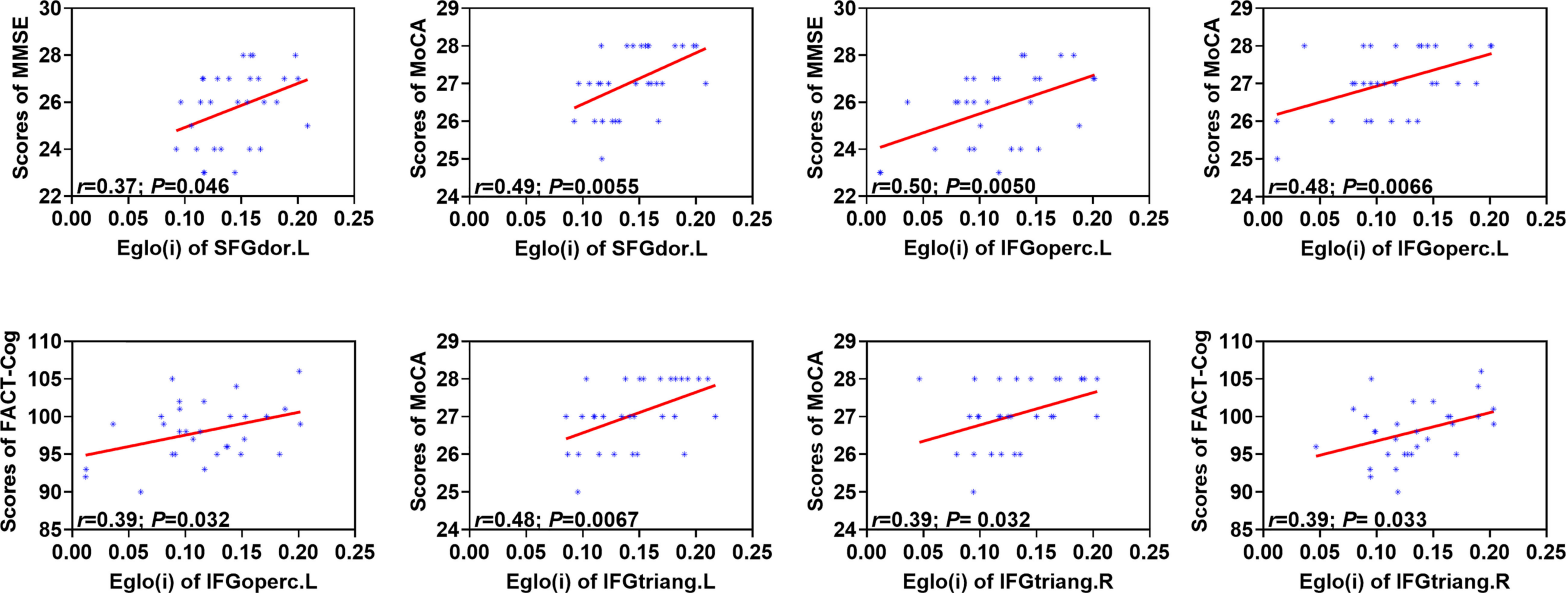
Associations between altered nodal global efficiency and cognitive assessment scores in the patient group. MMSE, Mini Mental State Exam; MoCA, Montreal Cognitive Assessment; FACT-Cog, Functional Assessment of Cancer Therapy-Cognitive Function. E_glo(i)_, nodal global efficiency. SFGdor.L, left superior frontal gyrus (dorsolateral); IFGoperc.L, left inferior frontal gyrus (opercular part); IFGtriang. L, left inferior frontal gyrus (triangular part); IFGtriang.R, right inferior frontal gyrus (triangular part). The associations were explored by the method of *Pearson’s* correlation and *P* < 0.05 was considered statistically significant.

As shown in **Figure 4**, E_glo(i)_ of the left superior frontal gyrus (dorsolateral) were positively correlated with the total scores of MMSE (*r*=0.37; *P*=0.046) and MoCA (*r*=0.49; *P*=0.0055) in the patient group. E_glo(i)_ of the left inferior frontal gyrus (opercular part) showed positive associations with the total scores of MMSE (*r*=0.50; *P*=0.0050), MoCA (*r*=0.48; *P*=0.0066) and FACT-Cog (*r*=0.39; *P*=0.032). In addition, positive relationship was found between E_glo(i)_ of the left inferior frontal gyrus (triangular part) and the total scores of MoCA (*r*=0.48; *P*=0.0067). Moreover, E_glo(i)_ of the right inferior frontal gyrus (triangular part) were positively related to the total scores of MoCA (*r*=0.39; *P*= 0.032) and FACT-Cog (*r*=0.39; *P*= 0.033).

## Discussion

In this study, based on rs-fMRI data, we investigated the differences of small-world properties, network and nodal parameters of the functional brain network between RC patients at the end of chemotherapy treatment (on the day patients received the last dose of chemotherapy) and HCs by graph theory analysis. Relationships between topological parameters of impaired brain regions and the scores of cognitive scales were also explored. Decreased small-world parameter γ and network measure L_p_ were found in RC patients when compared with HCs. Additionally, abnormal E_glo(i)_ of the superior and inferior frontal gyrus were positively associated with the level of cognitive function in the patient group.

Chemotherapy-induced cognitive impairment was identified in survivors with non-central nervous system cancers administered with adjuvant chemotherapy (42, 43). The cognitive dysfunction includes impaired executive function, attention, working memory and learning, which are attribute to the central nervous system toxicity of chemotherapeutic agents (44). The cognitive impairment has been recognized in patients with non-central nervous system cancers who undergone chemotherapy treatment (45). In this study, we also found that RC patients had decreased scores on the scales of MMSE, MoCA and FACT-Cog on the day patients received the last dose of chemotherapy. The impaired cognition induced by chemotherapy might increase the level of anxiety about the cancer and treatment in cancer patients. However, the central neural mechanism underlying chemotherapy related cognitive impairment (‘‘chemo brain’) is still poor.

The method of rs-fMRI can provide information about the patterns of neural activity in the brain. Advanced neuroimaging techniques and neuropsychological evaluation provide a useful tool for detecting the brain functional or structural changes due to neurodegenerative pathologies, such as cognitive impairment. Therefore, it has the potential for clarifying the neural bases of the cognitive impairment in RC patients after chemotherapy treatment (46, 47). Currently, the widely recognized brain networks are the default mode network (DMN), salience network (SN), attention network (AN), frontoparietal network (FPN), sensory-motor network (SMN), visual network (VN) (48). Previous neuroimaging studies had demonstrated that a set of interrelated brain networks including DMN, AN and FPN were considered to play a vital role in the cognitive processing (49–51).

The DMN is deactivated during execution of attention demanding tasks (52). Many studies provided evidence of disease-related abnormalities in DMN and aberrant functional connectivity was found in the DMN of patients with cognitive deficits (53, 54). In this study, the topological characteristics of left superior frontal gyrus (dorsolateral) was found to be impaired in the functional brain network of RC patients. Moreover, altered topological measure of this brain region was associated with the impaired cognition of RC patients. The superior frontal gyrus (dorsolateral) is considered as a part of DMN, which plays an important role in the cognitive processing (55). Patients with cognitive decline showed deficits in the nodal shortest path of superior frontal gyrus (dorsolateral) and other frontal regions (56). In addition, the network parameter γ decreased significantly in patients with cognitive impairment and significantly associations were identified between the nodal parameters of the superior frontal gyrus (dorsolateral) and cognitive function scores in previous rs-fMRI study using graph theory (57). Therefore, we suspected that aberrations of DMN were associated with cognitive deficits of RC patients who had just completed chemotherapy treatment, which might be involved in the neuropathological mechanisms underlying the acute effects of chemotherapy on cognitive function.

In the present study, the small-world properties were not impaired in RC patients at the end of chemotherapy treatment. The brain networks with small-world features, which characterized densely local connections and few long connections, had economical properties with high efficiency of parallel information transfer with low cost (27). This finding suggested that the overall distribution and average information transfer efficiency of the whole brain network were not impaired in RC patients who had just completed chemotherapy. Therefore, both high functional segregation and high functional integration of the whole brain network were preserved after the treatment of chemotherapy. However, the left superior frontal gyrus (orbital part), inferior frontal gyrus (opercular part), inferior frontal gyrus (triangular part) and right inferior frontal gyrus (triangular part) exhibited abnormal E_glo(i)_ in the functional brain network of RC patients at the end of chemotherapy treatment. Both the impaired E_glo(i)_ of left inferior frontal gyrus (opercular part) and bilateral inferior frontal gyrus (triangular part) were related to the cognitive impairment of RC patients. The measure of E_glo(i)_ reflected the efficiency of the information transfer of the region within the whole brain network, which was associated with the functional integration of this brain region. Patients with cognitive impairment had selectively enhanced impairment of structural connectivity in the superior frontal gyrus and inferior frontal gyrus and decreased functional connectivity in the right superior frontal gyrus when compared with those without cognitive deficiency (58, 59). In addition, reduced functional connectivity density was identified in the left inferior frontal gyrus and superior frontal gyrus, and the functional connectivity of the left inferior frontal gyrus was positively correlated with MMSE scores in patients with cognition decline (60). These regions are parts of AN, which is referred to as the task-positive network and are activated in tasks demanding attention control (61, 62). The AN plays an important role in the attentional control and are associated with cognitive effort necessary to perform a task (63, 64). The activities of DMN and AN are anticorrelated and the brain is intrinsically organized into anticorrelated networks (65). Reduced functional anticorrelations between DMN and AN were found in patients with cognitive impairment (63). We suspect that impaired topological properties of regions in the AN may be also associated with cognitive deficits of RC patients who had just receive the last dose of chemotherapy. Moreover, the abnormalities of functional anticorrelations between DNM and AN might play an important role in the neural mechanisms underlying acute effects of chemotherapy on cognitive function in RC patients. However, it was less optimal that the differences of network measures were uncorrected, which showed no significant differences when the method of correction for multiple comparisons was applied. Therefore, the results of network measures should be confirmed in the further studies with larger sample size.

Several limitations needed to be further addressed in this study. Firstly, the differences between RC patients received chemotherapy and HCs might be associated with chemotherapy or other factors, such as cancer itself, cancer related stress and other pharmacological treatments, etc. Due to the limited samples resulted from the difficulty of sample collection, there were no enough samples for comparisons between subgroups at present. This problem would be explored in our further studies comparing the differences of brain function between patients with and without chemotherapy, as well as between patients with more vs less cycles. In addition, other factors, such as inflammation or disrupted pro-inflammatory circuits and stress-related processes might cause a cascade of inflammatory response throughout the brain, which could also explain the mechanisms underlying cancer-related acute cognitive impairement. Therefore, the relationships between inflamatory or stress-related processes and impaired brain function or cognitive impairement should also be explored in the further studies. Secondly, the relatively small sample size might reduce its power to detect differences between groups. The limited clinical information of subjects might restrict the statistical power for detecting the functional brain changes underlying cognitive decline. Finally, the abnormal topological characteristics of the functional brain network observed in this cross-sectional study should be interpreted with caution. Both the changes of brain function and cognitive function should be compared between patients just completed the last dose of chemotherapy and those 6 months and 2 years after completion of chemotherapy. This would help us further understand the pathological mechanisms underlying the transient and long-term effects of chemotherapy on brain function and cognitive function.

## Conclusion

RC patients after chemotherapy treatment (on the day patients received the last dose of chemotherapy) had impaired cognition and demonstrated abnormalities in functional brain network topology. The findings demonstrated that the functional brain network of RC patients at the end of chemotherapy treatment had preserved small-world topological features but with a tendency towards higher path length, which suggested decreased integration and transmission of information of the brain, especially for the superior and inferior frontal gyrus. These abnormalities of topological organization might reflect the neuropathological mechanisms underlying acute neurotoxicity effects of chemotherapy on cognitive function in RC patients who had just completed chemotherapy.

## Data availability statement

The raw data supporting the conclusions of this article will be made available by the authors, without undue reservation.

## Ethics statement

The studies involving human participants were reviewed and approved by the Medical Ethics Committee of Jiangsu Cancer Hospital & Jiangsu Institute of Cancer Research & The Affiliated Cancer Hospital of Nanjing Medical University. The patients/participants provided their written informed consent to participate in this study.

## Author contributions

SiL, YG, ShengL and XL designed the experiments. SiL, YG, FY, NY, JN, CL, XP, RM, JW and XL contributed to clinical data collection and assessment. SiL, YG, ShengL and XL analyzed the results. SiL, YG and ShengL wrote the manuscript. All authors contributed to the article and approved the submitted version.

## Funding

The work was supported by the grants of: Jiangsu Provincial Natural Science Fund (No. BK20210977); Cadre Health Research Project of Jiangsu Province (No. BJ18033) and Foundation of Jiangsu Cancer Hospital (No. ZM201923).

## Conflict of interest

The authors declare that the research was conducted in the absence of any commercial or financial relationships that could be construed as a potential conflict of interest.

## Publisher’s note

All claims expressed in this article are solely those of the authors and do not necessarily represent those of their affiliated organizations, or those of the publisher, the editors and the reviewers. Any product that may be evaluated in this article, or claim that may be made by its manufacturer, is not guaranteed or endorsed by the publisher.
