# Supplementary material for: Associations between disrupted functional brain network topology and cognitive impairment in patients with rectal cancer during chemotherapy
Source: Front Oncol. 2022 Nov 24;12:927771. doi: 10.3389/fonc.2022.927771 (PMC9731768; doi:10.3389/fonc.2022.927771)
Supplement: Supplementary file 1 [file DataSheet_1.docx]

**1. MRI data acquisition**

All MRI data were obtained with a 3.0 T Philips Aachieva scanner. T1-weighted images were acquired with the following parameters: repetition time (TR)/echo time (TE)=9/2.48ms; flip angle (FA)=9°; field of view (FOV)=200×200mm^2^; matrix=200×200; voxel size=1×1×1mm; slice gap=0; slice number =176; acquisition time=4min 24s. The rs-fMRI data were acquired with the following parameters: TR/TE=3000/40ms; FA=90°; FOV=240×240mm^2^; matrix=80×80; voxel size=3×3×4mm; slice gap=0; slice number=34; acquisition time=6min 48s.

**2. MRI data preprocessing**

MRI data were preprocessed using the Data Processing Assistant for rs-fMRI advanced edition (DPARSF) based on MATLAB and SPM. The standard preprocessing steps were as follows: (1) the first 6 volumes were discarded for signal stabilization; (2) time differences in slice acquisition and head motion were corrected; (3) realignment to the middle image; (4) spatial normalization to the Montreal Neurological Institute (MNI) template; (5) resampled to 3×3× mm^3^; (6) a 4-mm full-width half-maximum (FWHM) Gaussian kernel for spatial smoothing is applied; (7) signal linear detrending was performed; (8) filtered with a temporal band-path of 0.01-0.1 Hz to reduce the low-frequency drift and high-frequency respiratory and cardiac noise; (9) nuisance covariates including the white matter signal, cerebrospinal fluid signal and Friston 24 motion parameters were regressed out. Participants were excluded if the head motion exceeded 2 mm of translation or 2° of rotation in any direction.

**3. Construction of functional brain network**

A network can be constructed by defining nodes and estimating edges. The whole brain was firstly parcellated into 90 regions of interest (ROIs) by the Automated Anatomical Labeling (AAL) atlas and each of the ROI was considered as a node in the functional brain network. *Pearson’s* correlations between the time series of all ROIs were extracted and were transformed into z-scores via *Fisher’s* transformation, which were defined as the connection strength of edges in the functional brain network. Finally, functional connectivity matrix was obtained and then undirected weighted network was constructed.
